# A beginning of the end: new insights into the functional organization of telomeres

**DOI:** 10.1080/19491034.2015.1048407

**Published:** 2015-05-11

**Authors:** Ashley M Wood, Kyle Laster, Ellen L Rice, Steven T Kosak

**Affiliations:** Department of Cell and Molecular Biology; Feinberg School of Medicine; Northwestern University; Chicago, IL, USA

**Keywords:** aging, chromosome structure, chromosome looping, genome stability, nuclear lamina, telomere

## Abstract

Ever since the first demonstration of their repetitive sequence and unique replication pathway, telomeres have beguiled researchers with how they function in protecting chromosome ends. Of course much has been learned over the years, and we now appreciate that telomeres are comprised of the multimeric protein/DNA shelterin complex and that the formation of t-loops provides protection from DNA damage machinery. Deriving their name from D-loops, t-loops are generated by the insertion of the 3′ overhang into telomeric repeats facilitated by the binding of TRF2. Recent studies have uncovered novel forms of chromosome end-structure that may implicate telomere organization in cellular processes beyond its essential role in telomere protection and homeostasis. In particular, we have recently described that t-loops form in a TRF2-dependent manner at interstitial telomere repeat sequences, which we termed interstitial telomere loops (ITLs). These structures are also dependent on association of lamin A/C, a canonical component of the nucleoskeleton that is mutated in myriad human diseases, including human segmental progeroid syndromes. Since ITLs are associated with telomere stability and require functional lamin A/C, our study suggests a mechanistic link between cellular aging (replicative senescence induced by telomere shortening) and organismal aging (modeled by Hutchinson Gilford Progeria Syndrome). Here we speculate on other potential ramifications of ITL formation, from gene expression to genome stability to chromosome structure.

## Introduction

Telomeres protect the ends of linear eukaryotic chromosomes, preventing them from degradation or recognition by DNA damage machinery. Telomerase activity and the shelterin complex regulate telomere length and stability. Telomerase is a ribonucleoprotein enzyme consisting of a reverse transcriptase (hTERT) and an associated template RNA (TERC), which function together to synthesize telomeric DNA. The multimeric shelterin complex protects telomeres by preventing the activation of DNA damage response pathways.^1^ Telomere-repeat binding factors (TRFs) 1 and 2 are core components of the shelterin complex that bind double-stranded telomeric DNA repeats. The primary role of TRF1 is thought to be in the regulation of telomere length,^2,3^ whereas TRF2 is thought to promote a protective telomere state.^4,5^ TRF2 likely carries out this function by facilitating the formation of a unique structure that telomeres use to disguise their free ends.^6,7^ This structure involves the insertion of the 3′ overhang at the end of chromosomes into telomeric DNA, forming loop structures known as t-loops.^8^ T-loops have been visualized both *in vitro* and *in vivo* and in a variety of organisms.^9-11^ TRF2 has also been shown to bind at internal genomic sites, mostly at TTAGGG repeat sequences referred to as interstitial telomeric sequences (ITSs).^12,13^ Loss of TRF2 function leads to increased activity of the DNA-damage response pathway, end-to-end chromosome fusion, and cellular senescence.^1,14-17^

**Figure 1.**
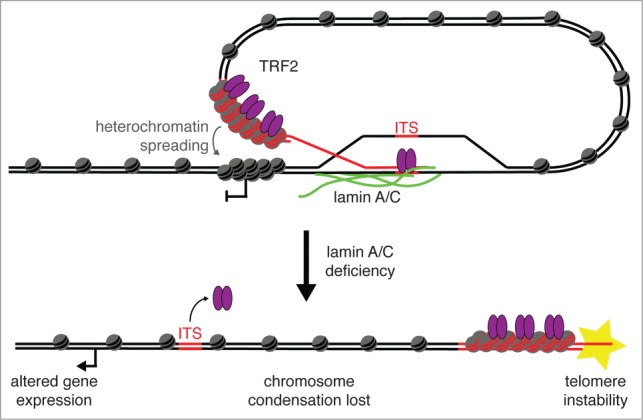
Model of ITL formation. Telomeric DNA (red) associates with ITSs found within non-telomeric DNA (black) to form ITLs. This association is facilitated by an interaction between TRF2 and lamin A/C and may result in heterochromatin spreading and gene inactivation in neighboring regions. In lamin A/C deficient cells (after lamin A/C knockdown or in progerin expressing cells), TRF2 no longer associates with ITSs resulting in a loss of ITL. This may result in altered chromatin state, misregulation of gene expression, loss of chromosome condensation, and telomere instability.

As described above, t-loops are TRF2-dependent loop structures that occur within the telomere itself. In our recently published work, we find evidence for a novel chromosome-end structure that involves telomeres interacting with DNA located outside of the telomere, forming a long-range chromosome loop that encompasses several megabases of chromatin (**Fig. 1**).^18^ As with t-loop formation, we find that this novel chromosome-end structure is TRF2 dependent. We find evidence for this structure in mitotic chromosomes from multiple human and mouse cell types as well as in human interphase nuclei, suggesting that this is a highly prevalent chromosome-end structure. We provide evidence that this chromosome loop structure involves an interaction between telomeres and ITSs, and we termed these structures interstitial telomeric loops (ITLs). Here we discuss the implications of ITL formation in organismal aging, telomere and genome stability, regulation of gene expression, and chromosome condensation.

## Lamin A/C and Telomere Protection

We found that in addition to TRF2 dependency, ITL formation requires lamin A/C, a critical component of the nuclear lamina. The nuclear lamina is a proteinacious network underlying the inner nuclear membrane and dispersed throughout the nucleoplasm. As an integral component of the nucleoskeleton, the nuclear lamina functions in many nuclear activities, including DNA replication, transcription, and chromatin organization.^19,20^ The core building blocks of the nuclear lamina are type V intermediate filament proteins, of which there are 2 classes: the A-type lamins (lamin A and C, encoded by *LMNA*) and the B-type lamins (lamin B1 and B2, encoded by *LMNB1* and *LMNB2*). A- and B-type lamins form distinct networks in the nucleus and each is thought to have a specific role in regulating gene expression and organizing chromatin.^21^ Interestingly, there is a lot of evidence for a link between lamin A/C and telomere stability, although the molecular and mechanistic connection was unclear prior to our recent work.

Much of what is known about the relationship between lamin A/C and telomeres comes from a premature aging disorder, Hutchinson Gilford Progeria Syndrome (HGPS). This disease is most often caused by a mutation in *LMNA* that results in expression of a permanently farnesylated form of the protein, called progerin. At the cellular level, HGPS leads to many defects including nuclear shape abnormalities, impaired mechanotransduction, loss of heterochromatin, and changes in gene expression.^22^ Furthermore, fibroblasts isolated from patients with HGPS exhibit reduced replicative capacity relative to age-matched controls,^23^ and human fibroblasts overexpressing wild-type or progeria-associated *LMNA* mutations also exhibit proliferation defects.^24,25^ Interestingly, these proliferation defects can be rescued by expression of hTERT,^25^ suggesting that in addition to the previously mentioned cellular phenotypes, a critical detrimental effect of disruption of the lamin A/C network is perturbation of telomere homeostasis. In agreement with these results, shortened telomeres are observed in fibroblasts isolated from HGPS patients compared to age-matched controls,^23,26^ in fibroblasts overexpressing progerin or wild-type lamin A/C^24^, and in mouse embryonic fibroblasts (MEFs) derived from *LMNA* deficient mice.^27^ Furthermore, evidence suggests that lamin A/C plays a role in the DNA damage response (DDR) pathway,^27-30^ and that progerin expression leads to DNA damage at telomeres that can be rescued by hTERT expression along with the rescue of cell proliferation defects mentioned previously.^25,31^ These results suggest that telomere instability is at least in part responsible for the reduced replicative capacity of cells with disrupted lamin A/C.

Despite all of these data that imply a connection between lamin A/C and telomere stability, the key molecular link behind this functional interaction remained to be uncovered prior to our recent study. We found that lamin A/C interacts with TRF2 and that the interaction is necessary for the association of TRF2 with ITSs. Additionally, we observed that progerin cannot interact with TRF2 and that a reduction in the frequency of ITL formation either after lamin A/C knockdown or in HGPS patient cells correlates with dramatic telomere instability. We therefore propose that the lamin A/C-TRF2-ITS interaction is critical for ITL formation and that the ITL chromosome-end structure masks telomeres functioning as an additional mechanism of telomere protection to those previously described. Our work does not rule out the possibility that lamin A/C regulates telomere stability through classic mechanisms of telomere protection, and further work is necessary to determine if t-loops and shelterin complex formation are affected by loss of lamin A/C function.

In addition to the nuclear lamina defects associated with premature aging in HGPS, normal aging also results in nuclear lamina abnormalities and expression of progerin.^32^ Therefore, we predict that disruption of the interaction between lamin A/C and TRF2 in normal, aged cells may play a role in aging at the organismal level. For example, lamin A/C is upregulated upon differentiation of human embryonic stem cells (hESCs) when telomerase expression is lost and cellular aging commences.^33,34^ Thus, lamin A/C may be involved in regulating cellular lifespan through ITL-facilitated telomere protection. Indeed, we find that TRF2 expression is concomitantly upregulated with lamin A/C upon loss of pluripotency of hESCs (data not shown). Analysis of ITL in hESCs and in the presence of telomerase will provide further insight into the relationship between chromosome-end structure and cellular aging. It is possible that the structure formed by ITL is only necessary for telomere protection when telomerase-mediated telomere maintenance is inactive. Future work will test this possibility and continue to clarify the link between cellular and organismal aging.

## Genome Stability

**Figure 2.**
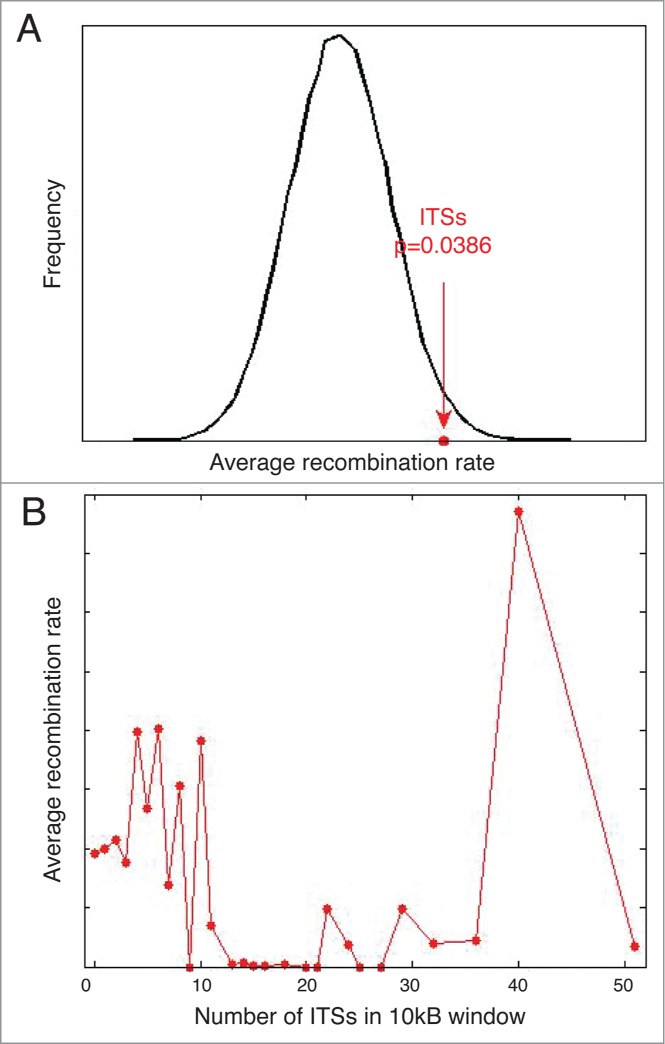
Small ITS are recombination hot spots. (**A**) The average recombination rate at small ITSs (red dot) is higher than expected compared to sites placed randomly throughout the genome (black line). Recombination rates were taken from Kong *et al.*^38^ (**B**) There is no correlation between the number of ITSs in a 10kb genomic window and the frequency of recombination.

In addition to the protective role that ITL formation appears to play at telomeres, there is also potential for increased genome instability if this chromosome-end structure is not properly stabilized. Large regions of repetitive telomeric repeats that can be found at internal genomic sites have been shown to correlate with fragile sites and hot spots for recombination.^35-37^ In order to determine if only large blocks of telomeric DNA associate with recombination, we asked whether very small ITSs, for these purposes defined as 2 TTAGGG repeats separated by less than 100bp, also show a correlation with increased recombination frequency.^38^ We determined that these small ITSs reveal an enriched frequency of recombination genome-wide (*P* < 0.04) (**Fig. 2A**). Intriguingly, we do not see a significant correlation between the density of ITSs and the frequency of recombination (**Fig. 2B**). This result suggests that isolated ITSs are just as likely to show recombination as more dense ITS regions and that it is not necessarily the occurrence of repetitive DNA that influences recombination, but the presence of certain ITSs. Since these small ITSs are highly prevalent throughout the genome, we find >25,000 sites genome-wide, it is not the appearance of this DNA sequence alone that stimulates ITL formation. Our work suggests that ITL formation at a given ITS is regulated by additional factors, in particular lamin A/C association. We speculate that lamin A/C mediated ITL formation disrupts normal chromatin formation and under certain conditions this structure may lead to increased rates of homologous recombination. Therefore, this correlation between small ITSs and recombination hot spots may be useful in identifying ITSs that are used for ITL formation *in vivo*.

The increased frequency of recombination at small ITSs may provide insight into the mechanism of ITL formation. Expression of a mutant form of TRF2 was shown to induce homologous recombination at traditional t-loops resulting in circular, telomere-containing DNA.^39^ This process results in telomere shortening at a rate that is far more rapid than replication-induced telomere shortening. If ITL formation can lead to homologous recombination in the same manner as traditional t-looping,^39,40^ this implies that the structures are similar and suggests ITL formation involves base pairing between the 3′ end of telomeric DNA and ITSs in a manner that is stabilized by lamin A/C (**Fig. 1**). Importantly, recombination at sites of ITL formation would result in a loss of large regions of genomic DNA in addition to telomeric DNA, and would therefore be extremely detrimental to the cell. This potential deleterious effect of ITL formation highlights the importance of understanding this novel chromosome structure in terms of genome instability.

## Regulation of Gene Expression

We propose that ITL formation involves the interaction of repressed, telomeric DNA with normal chromatin. This model raises intriguing questions about chromatin state and gene expression at sites of telomere interaction. An effect from ITL would be distinct from telomere position effect (TPE), which involves the spreading of heterochromatin linearly from telomeric DNA into neighboring subtelomeric regions.^41^ Interestingly, although it is still unclear whether yeast form an authentic t-loop, characterization of yeast telomeres has suggested that they form a fold-back structure that can occur into non-telomeric DNA in a manner that can regulate gene expression.^42-44^ These data from yeast support the argument that looped telomeric DNA can affect the surrounding chromatin, and a recent study by Robin *et al.* 2014 provides evidence that this may occur in human cells as well.^45^

Robin *et* al. 2014 reported interactions between subtelomeric regions and intrachromosomal DNA several megabases away, suggestive of long range, chromosome-end looping similar to what we have proposed as ITL. Interestingly, this study shows loss of looping upon replication-induced telomere shortening that is accompanied by changes in gene expression. They have called this phenomenon TPE over long distances (TPE-OLD). These findings suggest that beyond telomere stability, chromosome-end structure may play a critical role in regulation of gene expression. This study identifies a minimum of 144 genes within 10Mb of telomeres that show TPE-OLD, though it is not clear how many of these genes participate in telomere looping themselves and which experience regulation from downstream effects. Furthermore, it is still unclear mechanistically how long-range telomere looping affects gene expression. Accumulation of heterochromatin protein 1 (HP1), part of the repressive machinery commonly found at telomeres, is not seen at affected genes.^45^ However, TRF2 is found at promoters of these genes and a loss of TRF2 association is observed in cells with shortened telomeres. These results parallel our work, which demonstrates that TRF2 is necessary to facilitate long-range telomere looping. Further analysis is necessary to determine whether TRF2 has a direct role in the regulation of gene expression at these promoters or if its role is indirect through the regulation of telomere looping.

If ITL can effect gene expression, this raises new possibilities for transcriptional regulation. There are many ITSs found throughout the genome and TRF2 association with these sites seems to be cell-type specific and cannot be predicted based on sequence alone.^12,13^ This implies that ITL formation is cell-type specific, and could therefore serve as a mechanism for cell-type specific regulation of gene expression. Furthermore, changes in ITL formation during cellular aging may have a role beyond telomere protection in that reduced ITL formation may alter gene expression patterns that influence cell fitness and replicative capacity.

## Chromosome Condensation During Cell Division

In addition to telomere protection and regulation of gene expression, the process of long-range chromosome-end looping has interesting implications for chromosome condensation (**Fig. 1**). Our model for ITL formation suggests that this structure involves telomere interaction with ITSs. However, ITSs are found throughout the genome, and it is unclear which of these sites are used for ITL formation. Since the choice of ITS would influence chromosome loop size, it is tempting to speculate that this could affect the degree of chromosome condensation. Interestingly, while we observe an inverted chromosome-end structure suggestive of ITL formation in a very high percentage of mitotic chromosomes (60–95%), a specific ITS only shows co-localization with the telomere in about 7% of interphase nuclei. Therefore, it is possible that ITS use is dynamic and when looking at a specific ITS at the single cell level, we only see a subset of the ITL formation that actually exists. A further component of this discrepancy could be that ITL formation may be more prevalent in mitosis than interphase due to an increased need for chromosome condensation.

**Figure 3.**
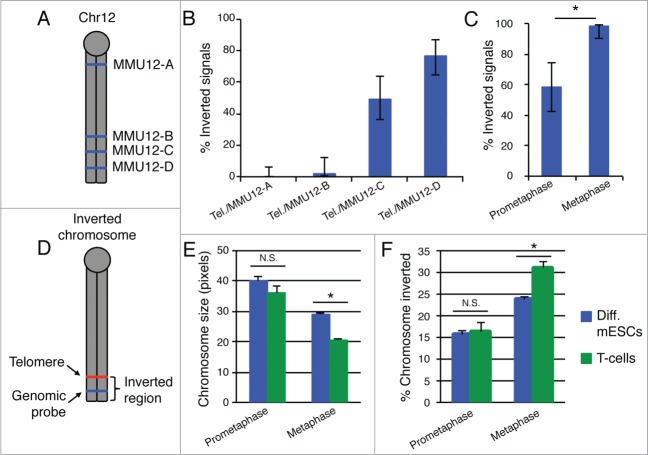
ITL may facilitate chromosome condensation. (**A**) A schematic of the location of genomic FISH probes used for ITL analysis on mouse chromosome 12. (**B**) The frequency of inverted telomeric/genomic FISH signals, as depicted in D, for genomic probes shown in A. The inversion frequency decreases with increasing distance from the chromosome end. Analysis was performed on mitotic chromosomes from differentiating mESC. Error bars represent 95% confidence intervals. (**C**) Metaphase chromosomes show a higher frequency of inverted telomeric/MMU12-D FISH signals than prometaphase chromosomes. Analysis was performed on mitotic chromosomes from differentiating mESCs. Error bars represent 95% confidence intervals. *P < 0.001, Student's t-test. (**D**) A schematic of an inverted chromosome structure. (**E**) Metaphase, but not prometaphase chromosomes isolated from differentiating mESCs are longer than those from T cells. Values represent mean ± s.e.m. *P < 0.001, Student's t-test. (**F**) The inverted region of the chromosome was determined by measuring the distance from a telomere FISH signal to the end of the chromosome, as shown in **D**. This value was reported as a percent of the total chromosome length. T cells show a larger region of inversion than differentiating mESCs for metaphase, but not prometaphase chromosomes. Values represent mean ± s.e.m. *P < 0.001, Student's t-test.

In order to further our understanding of the position of ITL formation in mitotic chromosomes, we used fluorescent *in situ* hybridization (FISH) probes located throughout mouse chromosome 12 and looked for the frequency of chromosome-end inversion (**Fig. 3A**). In this assay, we compare the position of a specific genomic probe to a probe that recognizes the telomere. If the telomeric probe is observed closer to the centromere, this represents an inverted chromosome structure that is suggestive of ITL formation (**Fig. 3D**). This analysis shows a decrease in inverted chromosomes with increasing genomic distance from the chromosome-end indicating that the telomere interacts more often with ITSs closer to the end of the chromosome (**Fig. 3B**). However, comparing MMU12-C and MMU12-D, there is a decrease in inverted signals, but not a clear cutoff as one would expect if the same ITS was used to facilitate ITL in every chromosome. Therefore, these data imply that there is variability in the position of ITL formation and are consistent with a model that uses ITS choice to influence the degree of mitotic chromosome condensation.

To more directly test for a role of ITL formation in chromosome condensation, we assayed ITL frequency at different stages of mitotic chromosome condensation. For this analysis, we compared prometaphase and metaphase chromosomes and found that ITL formation is more prevalent in the more condensed, metaphase chromosomes (**Fig. 3C**). Additionally, we compared chromosomes from differentiating mouse embryonic stem cells (mESCs) and a murine T cell line (EL-4). Metaphase chromosomes from T cells are substantially smaller than those of differentiating mESCs, and although in a similar trend, prometaphase chromosomes do not show a significant difference (**Fig. 3E**). Comparing these 2 cell types, we found little difference in prometaphase chromosome ITL, but observed that the more compact, T-cell metaphase chromosomes showed a significantly larger region of the chromosome involved in looping than differentiating mESC metaphase chromosomes (**Fig. 3D & F**). Together, these results suggest that ITL formation may play a role in progressive chromosome condensation during mitosis through the use of ITSs at increasing distances from the chromosome end. In addition to decreased chromosome size, T cells also have smaller interphase nuclei than differentiating mESCs, which raises intriguing implications about the relationship between chromosome condensation and nuclear size. Therefore, how this potential chromosome condensation function of ITL formation translates to interphase nuclei is a noteworthy question, and it will be interesting to determine whether distinct ITSs are used during mitosis and interphase. Moreover, the potential for telomeres to dynamically access ITSs during cell division to facilitate chromosome condensation may manifest itself in the reciprocal translocations of spatially adjacent chromosomes. This will be an important avenue to pursue.

## Perspective

The identification of ITL as a previously undescribed chromosome-end structure has implications in telomere and genome stability as well as regulation of gene expression and chromosome condensation. Important, yet currently unanswered, questions involve understanding how various ITSs are chosen to form this chromosome loop, and the dynamics of loop formation during the cell cycle and upon cellular differentiation. Addressing these questions will involve a combination of molecular and cellular approaches to understand loop formation at the single cell level. We have just begun to explore this phenomenon of long-range chromosome-end looping, and future research on the mechanism and downstream effects of this chromosome structure are sure to unlock a wealth of information on chromosome form and function and its implications in aging and disease.

## Disclosure of Potential Conflicts of Interest

No potential conflicts of interest were disclosed. 
